# Molecular Cloning and Functional Characterization of a β-Glucosidase Gene to Produce Platycodin D in *Platycodon grandiflorus*

**DOI:** 10.3389/fpls.2022.955628

**Published:** 2022-07-04

**Authors:** Xinglong Su, Fei Meng, Yingying Liu, Weimin Jiang, Zhaojian Wang, Liping Wu, Xiaohu Guo, Xiaoyan Yao, Jing Wu, Zongping Sun, Liangping Zha, Shuangying Gui, Daiyin Peng, Shihai Xing

**Affiliations:** ^1^School of Pharmacy, Anhui University of Chinese Medicine, Hefei, China; ^2^Institute of Traditional Chinese Medicine Resources Protection and Development, Anhui Academy of Chinese Medicine, Hefei, China; ^3^College of Humanities and International Education Exchange, Anhui University of Chinese Medicine, Hefei, China; ^4^College of Life Sciences and Environment, Hengyang Normal University, Hengyang, China; ^5^Engineering Technology Research Center of Anti-aging, Chinese Herbal Medicine, Fuyang Normal University, Fuyang, China; ^6^Anhui Province Key Laboratory of Pharmaceutical Preparation Technology and Application, Anhui University of Chinese Medicine, Hefei, China; ^7^MOE-Anhui, Joint Collaborative Innovation Center for Quality Improvement of Anhui Genuine Chinese Medicinal Materials, Hefei, China; ^8^Anhui Province Key Laboratory of Research and Development of Chinese Medicine, Anhui University of Chinese Medicine, Hefei, China

**Keywords:** *Platycodon grandiflorus*, triterpenoid saponins, β-glucosidase, subcellular localization, functional characterization

## Abstract

Platycodin D (PD) is a deglycosylated triterpene saponin with much higher pharmacological activity than glycosylated platycoside E (PE). Extensive studies *in vitro* showed that the transformation of platycoside E to platycodin D can be achieved using β-glucosidase extracted from several bacteria. However, whether similar enzymes in *Platycodon grandiflorus* could convert platycoside E to platycodin D, as well as the molecular mechanism underlying the deglycosylation process of platycodon E, remain unclear. Here, we identified a β-glucosidase in *P. grandiflorus* from our previous RNA-seq analysis, with a full-length cDNA of 1,488 bp encoding 495 amino acids. Bioinformatics and phylogenetic analyses showed that β-glucosidases in *P. grandiflorus* have high homology with other plant β-glucosidases. Subcellular localization showed that there is no subcellular preference for its encoding gene. β-glucosidase was successfully expressed as 6 × His-tagged fusion protein in *Escherichia coli* BL21 (DE3). Western blot analysis yielded a recombinant protein of approximately 68 kDa. *In vitro* enzymatic reactions determined that β-glucosidase was functional and could convert PE to PD. RT-qPCR analysis showed that the expression level of β-glucosidase was higher at night than during the day, with the highest expression level between 9:00 and 12:00 at night. Analysis of the promoter sequence showed many light-responsive cis-acting elements, suggesting that the light might regulate the gene. The results will contribute to the further study of the biosynthesis and metabolism regulation of triterpenoid saponins in *P. grandiflorus*.

## Introduction

*Platycodon grandiflorus* (Jacq.) A. DC. is a perennial herb of the family Campanulaceae, the roots (*Platycodi radix*) of which are used as medicinal herbs in Northeast Asia for their curative effect on respiratory diseases, including bronchitis, tonsillitis, sore throat, asthma, and tuberculosis (Lee et al., 2012; Zhang et al., 2015). Oleanane-type triterpenoid saponins, especially platycodin D [formal name: 3β-(β-D-glucopyranosyloxy)-2β, 16α, 23, 24-tetrahydroxy-O-D-apio-β-D-furanosyl-(1 → 3)-O-β-D-xylopyranosyl-(1 → 4)-O-6-deoxy-olean-12-en-28-oic acid, PD], are the major active components in *P. grandiflorus*. PD exhibits a wide range of pharmacological effects, including anti-atherosclerosis (Wu et al., 2012), anti-obesity (Lee et al., 2012), anti-inflammation (Guo et al., 2021), anti-oxidant (Wang et al., 2018), anti-aging (Shi et al., 2020), and anti-cancer (Huang et al., 2019) effects, as well as possesses excellent application and development potential.

PD consists of a main oleanane-type backbone with C3-Glc and C28-O-Api-Xyl-Rha-Ara. In addition, platycoside E (PE) is a major platycoside, accounting for more than 20% content of the *platycodi radix* platycosides (Yoo et al., 2011). The chemical structure of PE includes only two more glucosyl groups (at the C3 position) than PD (Supplementary Figure 1). Leaves of *P. grandiflorus* are rich in PE, which were discarded when collecting medicinal plants. (Shin et al., 2019; Su et al., 2021; Yu et al., 2021). Reducing the PE content-a lot of which is wasted a lot in actual production-may increase the PD content of *P. grandiflorus* plants. The pharmacological activities of deglycosylated ginsenosides are considerably higher than those of glycosylated ginsenosides (Shin and Oh, 2016), owing to theirs’ lower molecular weight, better hydrophobicity, and easier absorption in the human gastrointestinal tract. Therefore, several studies have investigated the deglycosylation of platycosides using various methods. Biotransformation, especially, displays the highest selectivity and productivity. PD is a deglycosylated triterpene saponin, whereas PE is a glycosylated triterpene saponin.

β-glucosidases (β-D-glucoside glucohydrolase, EC 3.2.1.21) are involved in diverse cellular functions (e.g., they hydrolyze the glycosidic bonds of alkyl-, amino-, or aryl-β-D glucosides, cyanogenic glucosides, disaccharides, and short oligosaccharides), and are ubiquitous enzymes found in archaea, eubacteria, and eukaryotes (Bhatia et al., 2002; Ketudat Cairns and Esen, 2010). These enzymes are involved in glycolipids breakdown and glucose release in plants to meet their metabolic needs (e.g., for cell growth and wall remodeling; Chuenchor et al., 2008; Horikoshi et al., 2022). β-glucosidases also display a range of aglycone specificities, a few of which show almost absolute specificity for one sugar and one aglycone, while others accept a range of either glycones or aglycones, or both (Baglioni et al., 2021; Dziadas et al., 2021; Prieto et al., 2021). They have been extensively studied in the biotransformation of PE to PD. The enzymatic biotransformation of PE to PD *in vitro* has been demonstrated in various studies using β-galactosidase from *Aspergillus oryzae* (Ha et al., 2010), snailase (Li et al., 2012b), laminarinase (Jeong et al., 2014), β-glucosidase from *Aspergillus usamii* (Ahn et al., 2018), recombinant β-glucosidases from *Caldicellulosiruptor bescii* (Kil et al., 2019), and cytolase (Shin et al., 2020). We further speculated that there might be a deglycosylase in *P. grandiflorus*, which could also catalyze the conversion of PE into PD (Su et al., 2021). Although great progress has been made in the study of how enzymes catalyzed the conversion of PE to PD, little is known about the β-glucosidase gene with this function in *P. grandiflorus*. Therefore, the present study aimed to investigate the β-glucosidase gene from *P. grandiflorus* using complementary DNA (cDNA) cloning and *in vitro* enzymatic characterization. Our findings may provide new insights into the analysis and regulation of the PD biosynthetic pathway in *P. grandiflorus*.

## Materials and Methods

### Plant Materials

*Platycodon grandiflorus* plants used in this study were the same materials described by Su et al. (2021). The specimen of *P. grandiflorus* was deposited in the Herbarium of Anhui University of Chinese Medicine (depository number 20200705). Samples were collected at 6:00 am, 9:00 am, 12:00 am, 3:00 pm, 6:00 pm, 9:00 pm, and 12:00 pm, 3 days in a row and immediately stored in liquid nitrogen for subsequent total RNA isolation. Three biological replicates were performed for each sample.

### Total RNA Preparation, cDNA Synthesis and RT-qPCR

Total RNA was extracted using TRNzol Universal Reagent (Tiangen Biotech Co., Ltd., Beijing, China) according to the manufacturer’s instructions. Each total RNA sample was qualified and quantified using the ultramicro spectrophotometer DS-11 (DeNovix, Wilmington, Delaware, USA). cDNA was synthesized using the FastKing RT Kit (Tiangen Biotech Co., Ltd., Beijing, China). SuperReal PreMix Plus (Tiangen Biotech Co., Ltd., Beijing, China) was used for RT-qPCR on the LightCycle480 platform (Roche, Switzerland) to determine the mRNA transcriptional levels of the genes encoding β-glucosidase and β-amyrin synthase (β-AS, GenBank: KY412556.1) in *P. grandiflorus*. The mRNA of *18sRNA* was used as an internal reference. Three biological replicates were used, and the relative expression of the mRNA level was calculated using the 2^−ΔΔCt^ method (Livak and Schmittgen, 2001). The sequence of the gene encoding β-glucosidase, its promoter sequence, and all the primer pairs used are listed in Supplementary Tables 1 and 2.

### Bioinformatics Analysis

Previously, the cDNA sequence of the candidate gene encoding β-glucosidase was obtained *via* transcriptomic sequencing (Su et al., 2021). ExPASy^1^ was used to deduct the amino acid (aa) sequences and predict physicochemical properties. The conserved domains of β-glucosidase were detected using Multiple Em for Motif Elicitation^2^ and the NCBI Conserved Domain Database (CDD)^3^ search tools. The possible transmembrane regions of the protein were analyzed using the TMHMM online program.^4^ PSORT^5^ was used to predict the subcellular localization of the protein encoded by the candidate gene. The secondary structure and three-dimensional homologous modeling of the protein were predicted using softwares of GOR IV,^6^ PDBsum,^7^ and SWISS-MODEL,^8^ respectively. Online PlantCARE^9^ was used to analyze the cis-acting elements of promoters. The amino acid sequence was used to perform homology searched on NCBI, and the sequences with high similarity were selected for multiple alignment. Phylogenetic analysis was performed using the MEGA X software (maximum likelihood), and using bootstrap analysis with 1,000 iterations.

### Subcellular Localization

The full-length cDNA sequence of the candidate gene encoding β-glucosidase without the terminator codons was inserted into the pBI121-EGFP vector to generate the pBI121-β-glucosidase-EGFP fusion protein, which, after sequencing, was subsequently transformed into *Agrobacterium tumefaciens* GV3101 using the freeze–thaw method. Two batches of *A. tumefaciens* containing pBI121-β-glucosidase-EGFP and pBI121-EGFP, respectively, was cultured overnight until the OD600 was approximately 1.0. Next, these batches of *A. tumefaciens* were resuspended in infiltration buffer (10 mM MgCl_2_, 10 mM MES (pH 5.6), and 100 μM acetosyringone) to an OD600 of 0.6 and incubated at room temperature for 2 h before being syringe-infiltrated into 5-week-old *Nicotiana benthamiana* leaves. Signals were observed under a ZEISS710 confocal laser scanning microscope (ZEISS, Germany) 48 h after infiltration.

### Preparation of Recombinant β-Glucosidase

β-glucosidase-3-F and β-glucosidase-3-R primers were designed to introduce new restriction sites by changing nucleotides (Supplementary Table 3). The target fragments were amplified using Ex Taq DNA polymerase (Takara, Biomedical Technology Co., Ltd., Beijing, China) *via* PCR, double-restricted using Bgl II/Xho I (NEB, Beijing, China), and ligated to the Bgl II/Xho I double-restricted pET-32ɑ(+) vector using T4 DNA Ligase (NEB, Beijing, China). The recombinant vector was transformed into *E. coli* DH5α cells using the freeze–thaw method, and positive transformants were selected on Lysogeny Broth (LB) agar plates containing ampicillin (final concentration: 50 μg/ml) for expansion and sequencing. *E. coli* BL21 (DE3) was then transformed with the recombinant plasmid for protein expression.

The transformed BL21 (DE3) cells were incubated in LB medium with 100 μg/ml ampicillin at 37°C until OD_600_ reached 0.6–0.8. After adding IPTG (isopropyl-β-D-thiogalactoside) at a final concentration of 0.3 mM to the medium, the cells were further cultured on a rotary shaker at 18°C for 18 h, then harvested and re-suspended in phosphate buffer (0.01 M, pH 7.2). Phenylmethyl sulfonyl fluoride (final concentration: 0.5 mM) and lysozyme (final concentration: 0.3 mg/ml) were added to the resuspension, followed by sonication at 4°C. The supernatant was collected *via* centrifugation, and the protein concentration was determined using the Modified BCA Protein Assay Kit (Shanghai Sangon Biotech, Shanghai, China). Proteins (20 μg) were separated using 12% SDS-polyacrylamide gel electrophoresis (SDS-PAGE) and visualized by staining with Coomassie Brilliant Blue R-250.

### Western Blot Analysis

Each protein sample was resolved separated using SDS-PAGE, transferred onto polyvinylidene fluoride (PVDF) membranes (Beijing Labgic technology Co., Ltd., Beijing, China), and blocked with 5% bovine serum albumin in TBST [20 mM Tris–HCl (pH 7.4), 150 mM NaCl, and 0.1% Tween 20] for 2 h at room temperature. The membrane was washed with TBST and incubated overnight at 4°C in a 1/3,000 dilution of anti-6 × His tag mouse monoclonal antibody (BBI life sciences, China). After three washes with TBST at 10 min intervals, the membrane was incubated in a 1/5,000 dilution of AP-conjugated rabbit anti-mouse IgG (BBI Life Sciences, China) at room temperature. After three washes with TBST, two washes with TBS [20 mM Tris–HCl (pH 7.4) and 150 mM NaCl], and one wash with AP buffer [100 mM NaCl, 50 mM Tris–HCl (pH 8.0), and 2 mM MgCl_2_], recombinant protein was visualized *via* BCIP/NBT alkaline phosphatase staining.

### *In vitro* Enzymatic Reactions

Four experimental groups were established to verify the function of putative β-glucosidase, namely, the crude enzymes from *E. coli* BL21 (DE3), the crude enzymes from *E. coli* BL21 (DE3) containing the pET-32ɑ(+) vector, the crude enzymes from *E. coli* BL21 (DE3) including the pET-32ɑ(+)-β-glucosidase recombinant vector, and boiled crude enzymes from *E. coli* BL21 (DE3) with the pET-32ɑ(+)-β-glucosidase recombinant vector. PE (final concentration: 0.4 mg/ml) and each crude enzyme extract (final concentration: 0.05 mg/ml) were mixed in a phosphate buffer (0.01 M, pH 7.2) and incubated at 37°C for 2 h. The reaction products were analyzed using the LC-16 high-performance liquid chromatography (HPLC) system (Shimadzu, Japan). A Topsil C18 column (4.6 mm × 250 mm; Agilent, USA) was used with the isocratic elution of water and acetonitrile (71:29). The flow rate was 1 ml/min with a detection wavelength of 210 nm, and the column compartment was maintained at 30°C (Su et al., 2021).

### Statistical Analysis

All experiments were performed independently at least three times, and the data are expressed as the mean ± standard error. GraphPad (version 8.0) suite was used for statistical analysis, and *p* ≤ 0.05 was deemed a statistically significant difference.

## Results

### Cloning and Analysis of the β-Glucosidase Encoding Gene

We previously obtained three candidate genes (CL4020.Contig1_All, Unigene 1627_All, and Unigene7900_All; Su et al., 2021). In the present study, we selected CL4020.Contig1_All with the highest similarity as the candidate gene of *Pgβ-glucosidase* for our investigation. The full length of the candidate β-glucosidase gene was amplified using the β-glucosidase-F/β-glucosidase-R primers (Supplementary Table 1), and the gene sequence was submitted to GenBank (OM867671) in NCBI. The resulting PCR products were subjected to 1% agarose gel electrophoresis, and the results are shown in Supplementary Figure 2. The full-length cDNA putatively encoding β-glucosidase is 1,488-base pair (bp)-long and encodes a polypeptide with 495 amino acids (aa). In addition, the molecular mass of β-glucosidase was found to be 56,479.22 Da, the theoretical isoelectric points were 5.16, and the half-life was estimated to be 30 h (mammalian reticulocytes, *in vitro*). The instability index (II) was computed to be 30.97 and was classified as stable. Conserved domain analysis revealed that the protein belongs to the glycosyl hydrolase family 1 (GH1), and the actual alignment was detected with superfamily member pfam00232 (Supplementary Figure 3). The predicted protein secondary domain shows that the protein amino acids mainly exist as alpha helices, extended strands, and random coils. Furthermore, according to the template 3gno.1.A model, the global model quality estimation (GMQE) value is 0.82 (Supplementary Figure 4). From the predicted results, the protein has no transmembrane regions.

### Phylogenetic Tree and Conserved Domain Analysis

**Figure 1:**
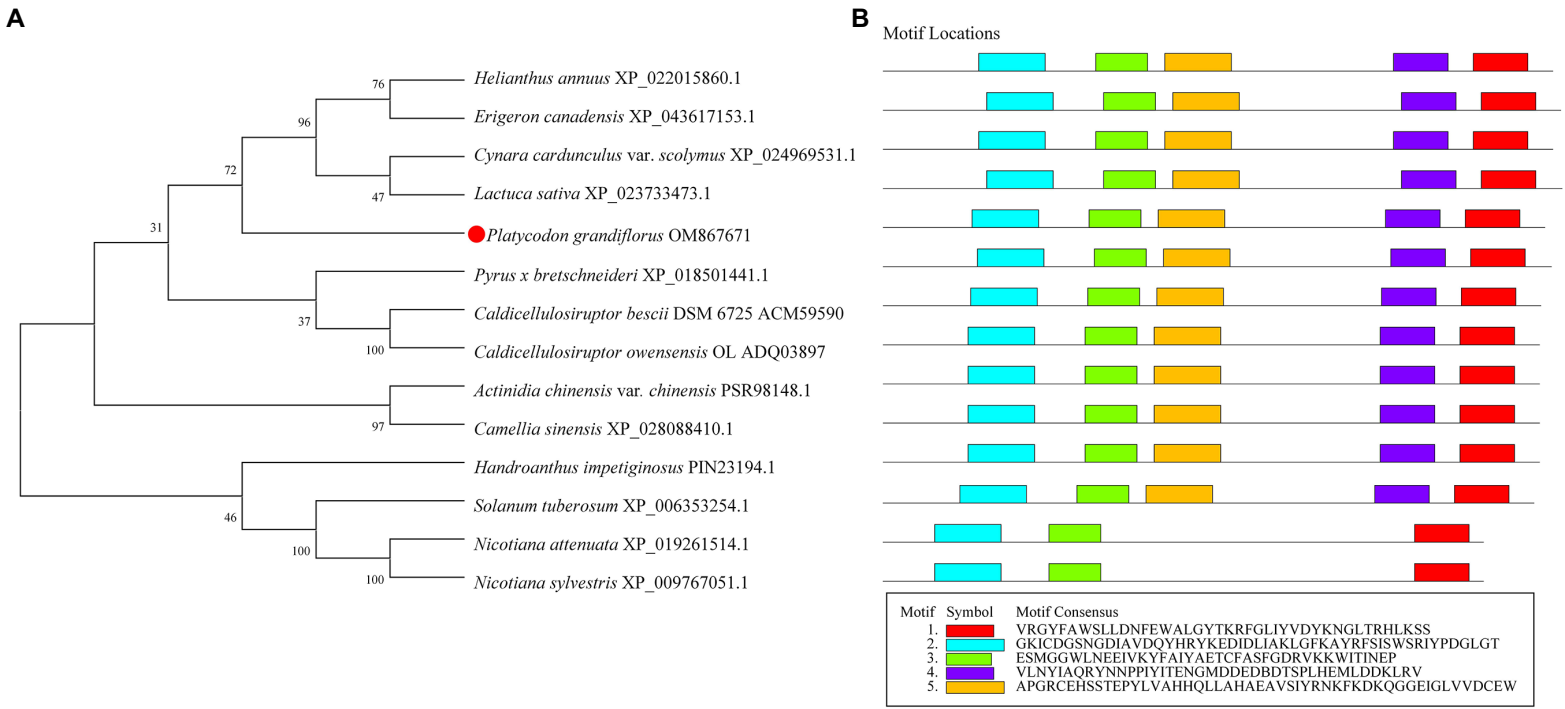
Phylogenetic and conserved domain analysis of Pgβ-glucosidase with those of other species. **(A)** Neighbor-joining phylogenetic tree, constructed using MEGA X. **(B)** Conserved domains, detected using Multiple Em for Motif Elicitation.

The phylogenetic tree of 12 different plants and two bacterial species was constructed, including *P. grandiflorus* (OM867671), *Nicotiana attenuata* (XP_019261514.1), *Nicotiana sylvestris* (XP_009767051.1), *Solanum tuberosum* (XP_006353254.1), *Handroanthus impetiginosus* (PIN23194.1), *Actinidia chinensis* var. *chinensis* (PSR98148.1), *Camellia sinensis* (XP_028088410.1), *Helianthus annuus* (XP_022015860.1), *Erigeron canadensis* (XP_043617153.1), *Cynara cardunculus* var. *scolymus* (XP_024969531.1), *Lactuca sativa* (XP_023733473.1), *Pyrus x bretschneideri* (XP_018501441.1), *C. bescii* DSM 6725 (ACM59590), and *Caldicellulosiruptor owensensis* OL (ADQ03897). Notably, β-glycosidases from *C. bescii* and *C. owensensis* have converted PE to PD (Kil et al., 2019; Shin et al., 2019). Compared with *P. grandiflorus*, the relationship between the β-glucosidase of *Pyrus x bretschneideri* and that of the two bacterial species was close. Four β-glucosidases (XP_022015860.1, XP_043617153.1, XP_022015860.1, and XP_023733473.1) from Asteraceae family members were phylogenetically closest to that of *P. grandiflorus* (Figure 1A). Furthermore, from the perspective of the evolutionary relationship of the genome, *H. annuus* is very close to *P. grandiflorus* and, they both belong to Asterids II (Kim et al., 2020).

**Figure 2:**
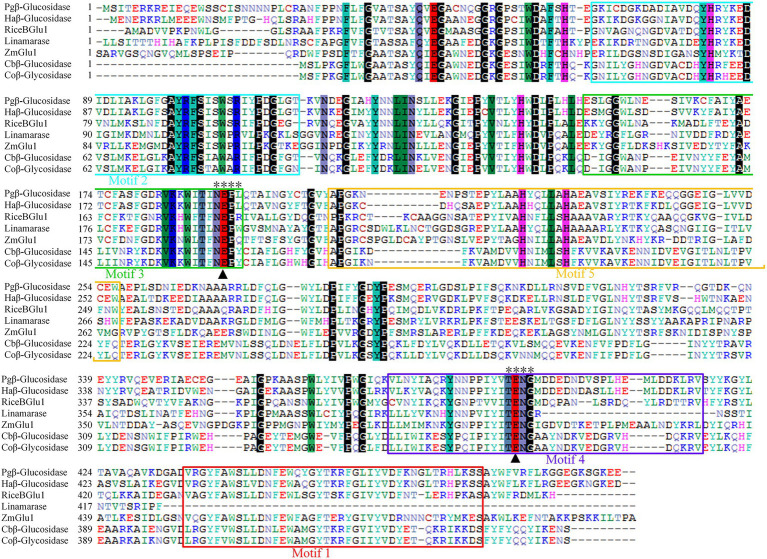
Amino acid sequences alignment of seven β-glucosidases. Black triangles indicate acid/base and nucleophilic catalytic residues. Two motifs (TENG and NEPX) are marked out with “*.” Pgβ-glucosidase, β-glucosidase of *Platycodon grandiflorus*; Haβ-glucosidase, β-glucosidase of *Helianthus annuus* (XP_022015860.1); RiceBGlu1, β-glucosidase of rice (PDB: 2RGL_A); Linamarase (GenBank: X56733.1); ZmGlu1, β-glucosidase of *Zea mays* (PDB: 1E1E_A); Cbβ-glucosidase, β-glucosidase of *Caldicellulosiruptor bescii* DSM 6725 (GenBank: ACM59590); Coβ-glucosidase, β-glucosidase of *Caldicellulosiruptor owensensis* OL (GenBank: ADQ03897).

Multiple sequence alignment revealed that Pgβ-glucosidase contained two conserved carboxylic acid residues (E192 and E393), serving as the catalytic acid–base and nucleophile (Henrissat et al., 1995; Jenkins et al., 1995; Chuenchor et al., 2008; Figure 2). These enzymes present a catalytic cycle that occurs in two distinct steps (glycosylation and deglycosylation), and where the two active sites involved are critical for their double displacement (Zechel and Withers, 2000; Marana, 2006; Chuenchor et al., 2011). Moreover, conserved domain analysis revealed that the β-glucosidases in other plants all possess motif 1, motif 2, motif 3, motif 4, and motif 5, except for those of *C. bescii* and *C. owensensis*, which only have motif 1, motif 2, and motif 3 (Figure 1B). However, β-glucosidase from *C. bescii* and *C. owensensis* have been shown to be function in catalyzing the conversion of PE to PD, thus, the common motif 1, motif 2, and motif 3 may have critical roles. In addition, E238, L298, and A425 in Pgβ-glucosidase were highly consistent with the β-glucosidases derived from *C. bescii* and *C. owensensis* and were different from other species (Figure 2).

### Subcellular Localization

**Figure 3:**
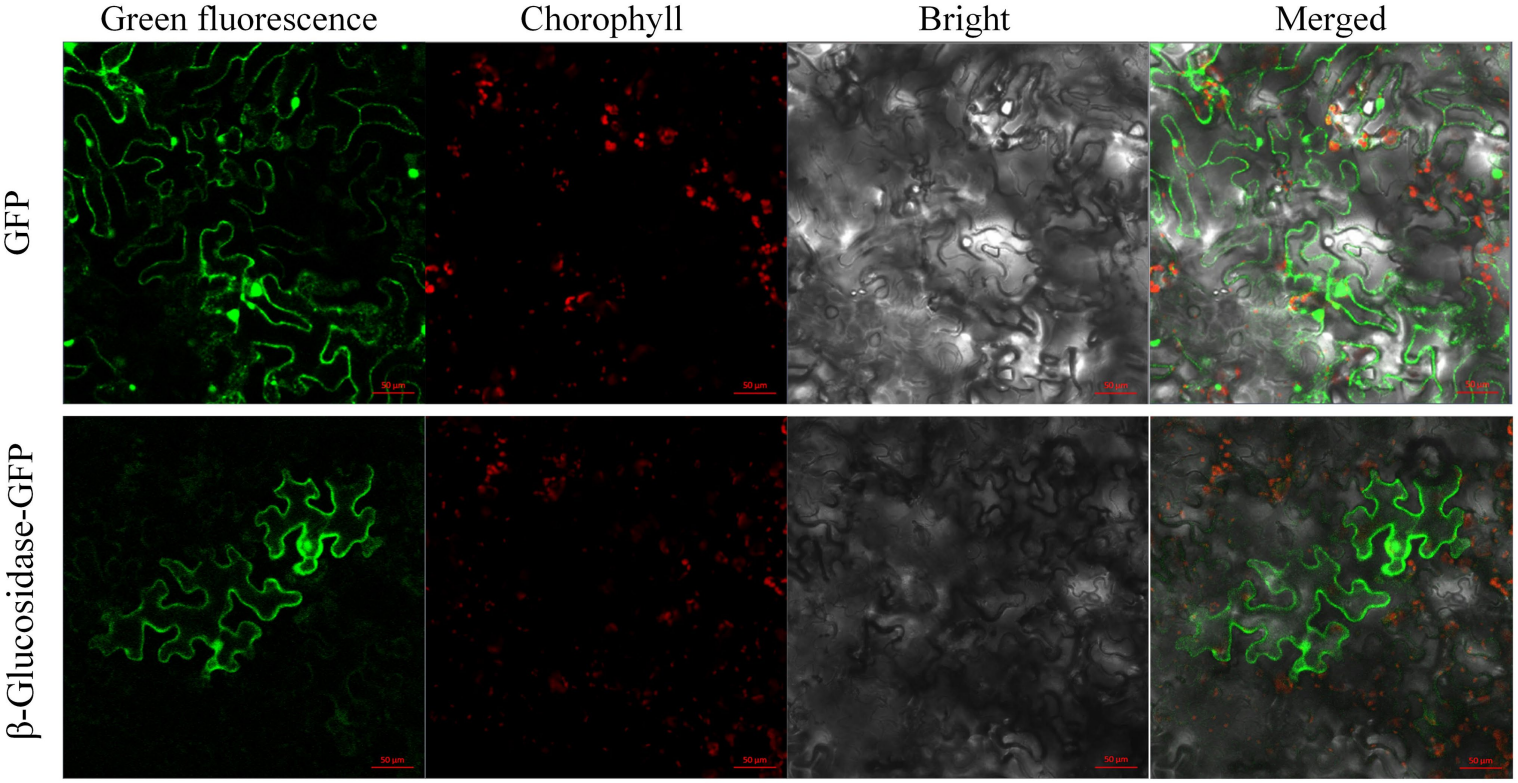
The subcellular localization of genes encoding Pgβ-glucosidase. The subcellular localization of 35S:: GFP, and 35S:: Pgβ-glucosidase-GFP in leaf epidermal cells of *N. benthamiana* leaf epidermal cells after 48 h infiltration; the epidermal cells of *N. benthamiana* were used for taking images of green fluorescence, chloroplast autofluorescence, visible light, and merged visible light.

Prediction of subcellular localization suggested that Pgβ-glucosidase was localized in the cytoplasm, mitochondria, and nucleus. GFP was fused to the C-terminal domain of the β-glucosidase protein and transiently transformed in *N. benthamiana* to determine the subcellular localization of β-glucosidase in *P. grandifloras*. Subsequently, confocal laser scanning microscopy was used to observe the *Agrobacterium*-infected the leaves of *N. benthamiana* containing the gene of interest, and the results are shown in Figure 3. Strong fluorescence signals were distributed in the cytoplasm and nucleus, consistent with the prediction. The results indicated that β-glucosidase was localized in the cytoplasm and nucleus of *P. grandiflorus*.

### Expression and Functional Assay of β-Glucosidase

**Figure 4:**
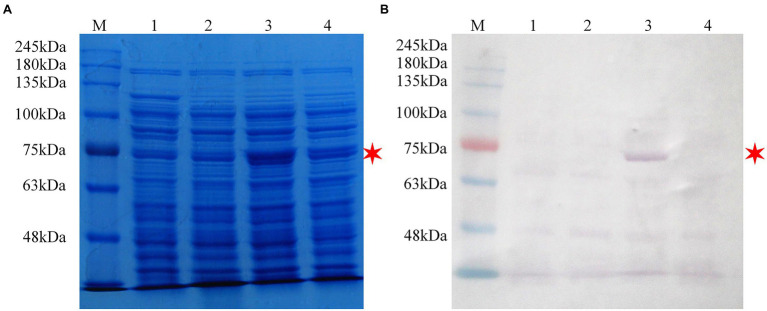
SDS-PAGE and western blot analysis of the recombinant protein pET-32ɑ(+)-Pgβ-glucosidase in *Escherichia coli* BL21 (DE3). **(A)** SDS-PAGE analysis. **(B)** Western blot analysis. M: protein molecular weight standards; Lane 1: cell lysate of *E. coli* BL21 (DE3; 0.3 M IPTG-inducing); Lane 2: cell lysate of *E. coli* BL21 (DE3)/pET-32ɑ(+; 0.3 M IPTG-inducing); Lane 3: cell lysate of *E. coli* BL21 (DE3)/pET-32ɑ(+)-Pgβ-glucosidase (0.3 M IPTG-inducing); Lane 4: cell lysate of *E. coli* BL21 (DE3)/pET-32ɑ(+)-Pgβ-glucosidase (1.0 M IPTG-inducing). Bacterial culture conditions were kept consistent.

Inducible expression of β-glucosidase recombinant protein was performed in pET-32ɑ(+) using Trx, a 6 × His and an S fusion tag at the N-terminal. With the size increase of 16.3 kDa by the fusion tags, the expected β-glucosidase protein was approximately 72.7 kDa (Figure 4). In addition to the expression of the recombinant plasmid (lane 3), *E. coli* BL21 (DE3) without the plasmid (lane 1) and *E. coli* BL21 (DE3) with the pET-32ɑ(+) vector (lane 2) were used as controls (Figure 4). In addition *E. coli* BL21 without the plasmid (lane 1) and *E. coli* BL21 with the pET-32ɑ(+) vector (lane 2) were performed as controls. Following crude protein quantification, 20 μg of proteins from each group was loaded separately and subjected to SDS-PAGE analysis. A thick band of recombinant β-glucosidase protein in lane 3 was in approximately 73.0 kDa (Figure 4A). Subsequently, western blotting using an anti-6 × His tag mouse monoclonal antibody showed a band of approximately 73.0 kDa on the PVDF membrane in lane 3, whereas lanes 1 and 2 (controls) showed no band. This finding confirmed the successful expression of β-glucosidase after transformation (Figure 4B).

**Figure 5:**
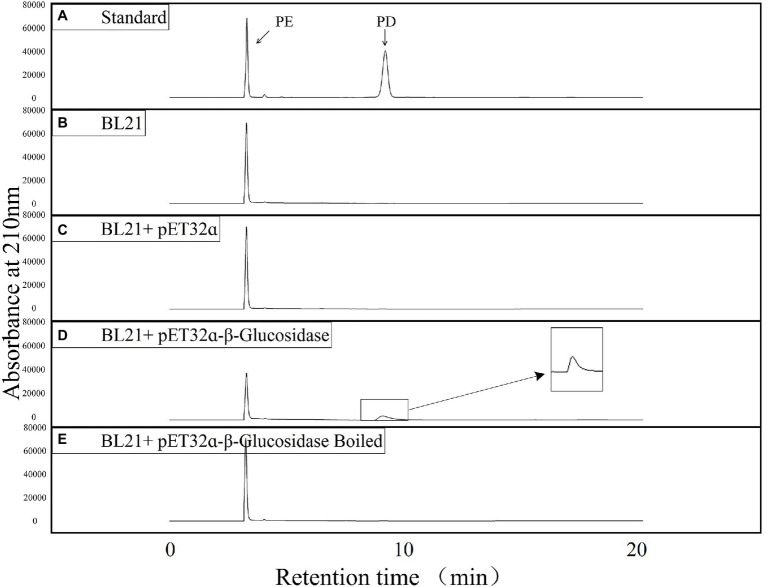
HPLC detection of the enzyme activities of putative Pgβ-glucosidase in producing PD. **(A)** Liquid chromatograms of standard PE and PD. **(B)** Liquid chromatogram of *E. coli* BL21 (DE3) cell lysate reacting with PE. **(C)** Liquid chromatogram of *E. coli* BL21 (DE3)/pET-32ɑ(+)cell lysate reacting with PE. **(D)** Liquid chromatogram of *E. coli* BL21 (DE3)/pET-32ɑ(+)-Pgβ-glucosidase cell lysate reacting with PE. **(E)** Liquid chromatogram of boiled *E. coli* BL21 (DE3)/pET-32ɑ(+)-Pgβ-glucosidase cell lysate reacting with PE.

The products of a putative conversion of PE to PD were analyzed using HPLC to verify the function of the candidate β-glucosidase. The reaction mixture, consisting of *E. coli* crude protein extracts (final concentration: 0.05 mg/ml) and PE (final concentration: 0.4 mg/ml) was incubated in a water bath at 37°C, and analyzed *via* HPLC after a 2 h reaction in 0.01 M phosphate buffer (pH 7.2). To test whether the β-glucosidase recombinant protein could catalyze the conversion of PE to PD *in vitro*, we incubated *E. coli* BL21 (DE3) protein extracts (BL21) and *E. coli* BL21 (DE3) with the pET-32ɑ(+) vector (BL21 + pET-32ɑ) under the same conditions. In addition, the experimental group (BL21-pET-32ɑ-β-glucosidase) protein was boiled to form a new control (BL21-pET-32ɑ-β-glucosidase boiled). Standard compounds of PD (> 98% purity) and PE (> 98% purity) were purchased from Chengdu Push Bio-Technology Co., Ltd., and the retention time and peak profile of 0.4 mg/ml of standard PD and 0.4 mg/ml of standard PE are shown in Figure 5A. Our results revealed that only the experimental group (BL21-pET-32ɑ-β-glucosidase) consumed PE and generated a new substance (PD; Figure 5). After excluding various effects of the control groups, we believe that recombinant target candidate β-glucosidase from *P. grandiflorus* can convert PE to PD *in vitro*.

### Analysis of β-Glucosidase Diurnal Expression

**Figure 6:**
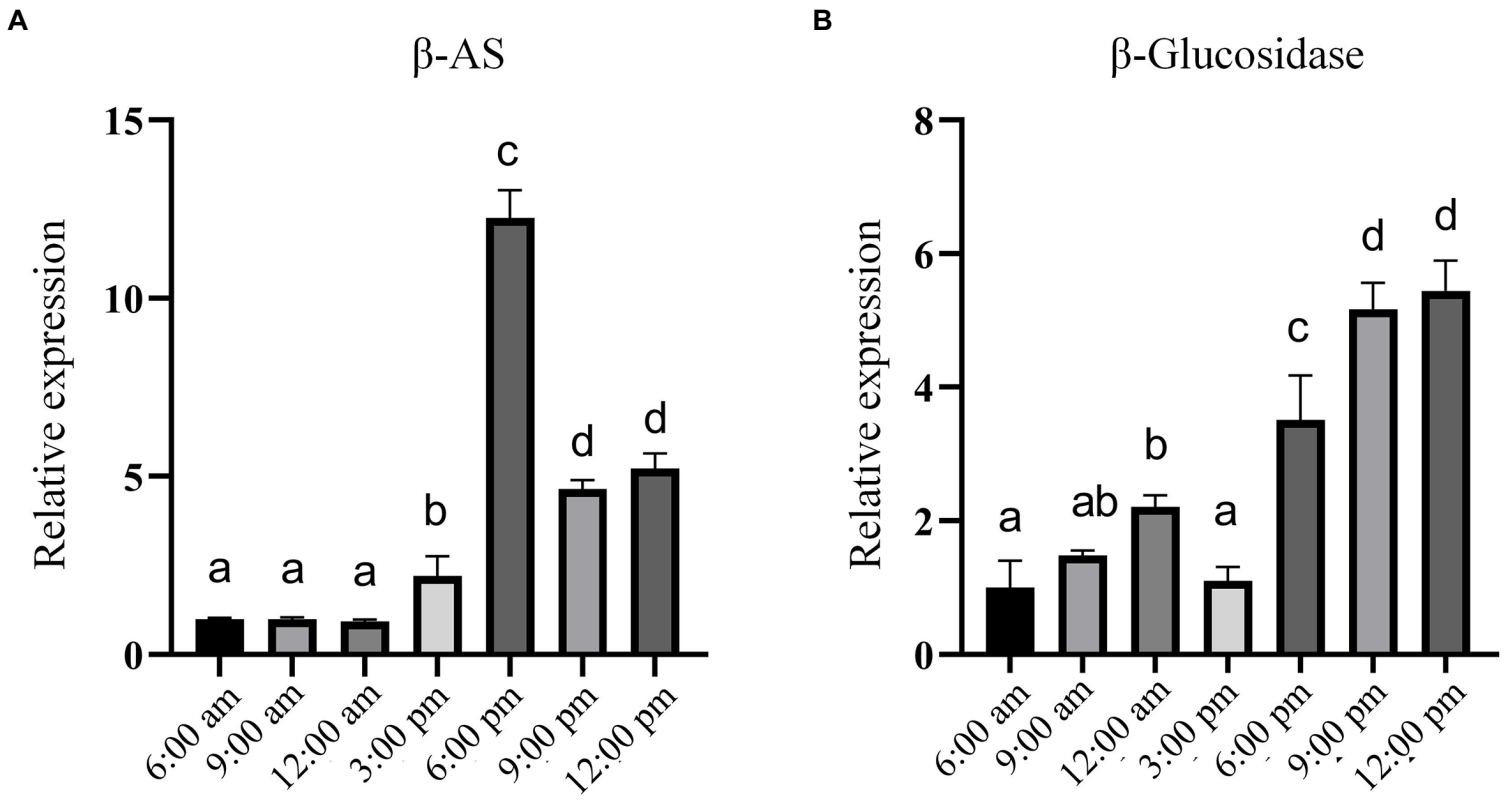
RT-qPCR analysis of the relative expression of genes encoding β-AS and Pgβ-glucosidase at different time points within 1 day. **(A)** Relative expression of *β-AS* gene. **(B)** Relative expression of the gene encoding Pgβ-glucosidase. Error bars indicate SD (*n* = 3) and different letters represent a value of *p* ≤ 0.05.

Studies have shown that the content of PE is higher in the leaves of *P. grandiflorus* than in other parts (Su et al., 2021; Yu et al., 2021). Therefore, we collected *P. grandiflorus* leaves at seven time points of the day and night from 6:00 am to 12:00 pm (at 3-h intervals) as samples for analysis. In addition, we simultaneously analyzed the expression of β-amyrin (β-AS), which is considered a key branching enzyme for generating oleanane-type triterpene backbones (Kim et al., 2020; Supplementary Figure 5). In other words, β-amyrin is a key enzyme in the biosynthetic pathway for synthesizing PE and PD in *P. grandiflorus*. The results of RT-qPCR analysis showed that the expression of β-amyrin remained unchanged from 6:00 am to 12:00  pm and then showed an upward trend. Furthermore, the expression was the highest at 6:00 pm and decreased after 6:00 pm; however, it was still higher than that during most times of the day (Figure 6). Similarly, the expression level of β-glucosidase is higher at night than during the daytime, with the highest values recorded from 9:00 to 12:00 at night. Furthermore, we obtained approximately 2000 bp of the promoter sequence (Supplementary Table 1) of the β-glucosidase gene from the *P. grandiflorus* genome (GenBank: GCA_016624345.1). Analysis of the promoter sequence of the β-glucosidase gene revealed the present of many light-responsive cis-acting elements such as AE-box, Box 4, GT1-motif, MRE, TCCC-motif, TCT-motif, and other cis-acting elements (Supplementary Figure 6). These results suggest that β-glucosidase gene expression may be regulated by light.

## Discussion

*Platycodon grandiflorus* is a common natural medicinal plant with a history of thousands of years that contains a wide variety of more than 80 chemical components, including saponins, alkynes, lipids, and flavonoids (Wang et al., 2017; Huang et al., 2021; Zhang et al., 2022). Modern pharmacological studies indicated that PD is one of the main active saponins of *P. grandiflorus* with broad bioactivities (Kwon et al., 2017; Zhang et al., 2017; Cho et al., 2018). The Chinese Pharmacopoeia also used PD content as the standard to measure the qualification of medicinal materials. In the present study, we identified a glycosyltransferase β-glucosidase from *P. grandiflorus* that can remove two glucose groups from PE to generate PD with stronger activity. Furthermore, the results of the phylogenetic analysis showed that Pgβ-glucosidase is the closest to *H. annuus* in the Compositae family, which is consistent with previous findings by Kim et al. (2020) on the evolutionary relationship of the *P. grandiflorus* genome. The subcellular localization results revealed that Pgβ-glucosidase is located in the nucleus and cytoplasm of *P. grandiflorus*. The results of the 1-day expression pattern and promoter sequence analysis indicated that the transition of related secondary metabolites might mainly occur at night; therefore, it is speculated that the gene encoding Pgβ-glucosidase may be regulated by light.

Analyzing known functionally related sequences for common sequence domains or motifs can reveal their association with a common function (Stormo, 2006). For example, the amino acid sequence of chalcone synthase from *Coelogyne ovalis* Lindl. shares four motifs with chalcone synthase from the *Oncidium* hybrid cultivar., *Cymbidium* hybrid cultivar., *Bromheadia finlaysoniana*, and *Dendrobium nobile*, which contains the active site “RLMLYQQGCFAGGTVLR” and the signature sequence of “GVLFGFGPGL” of the chalcone synthase protein (Singh and Kumaria, 2020). In addition, a study of β-glucosidase found that most of its homologous sequences contained the conserved TENG and NEPW motifs, which contained two conserved glutamic acid residues as acid/base catalyst and an active catalytic nucleophile, respectively (Zada et al., 2021a,b). We found five conserved motifs that also included the conserved TENG and NEPX motifs of β-glucosidases. The results of multiple sequence alignment revealed that E192 and E393 are highly conserved in the β-glucosidase sequences of seven creatures, including two bacterial species (*C. bescii* and *C. owensensis*). This finding suggests that Pgβ-glucosidase may also deglycosylate PE into PD through a double displacement reaction.

β-glucosidases primarily catalyze the removal of terminal non-reducing β-D-glucosyl residues from various glucoconjugates, including glucosides, oligosaccharides, and 1-O-glucosyl esters, as well as perform transglycosylation and reverse hydrolysis. Due to the extensive distribution of their substrates, these enzymes exist widely in nature and exhibit a range of functions (Godse et al., 2021). Studies have shown that β-glucosidase promotes the formation of free terpenes, phenylpropenes, and specific aliphatic esters during wine fermentation and promotes the production of wine aroma compounds that affect the aroma and flavor of the product (Liu et al., 2021). β-glucosidase was identified from mangrove soil showed high hydrolyzing ability for soybean isoflavone glycosides *via* heterologous expression in *E. coli*, which could completely convert daidzin and genistin to daidzein and genistein, respectively (Li et al., 2012a). Studies have also shown that β-glucosidase from the thermophilic fungus *Talaromyces leycettanus* JM12802 could hydrolyze isoflavone glycosides to aglycones (Li et al., 2018). Furthermore, recombinantly expressed β-glucosidase from *Sulfolobus solfataricus* and *Microbacterium esteraromaticum* transformed ginsenoside Rb1 to the more stable and readily absorbed, as well as more pharmacologically active, ginsenoside compound K and ginsenoside 20(S)-Rg3, respectively (Noh et al., 2009; Li et al., 2012a). β-glucosidase may also be an economically viable option for industrial use, with the production of pharmaceutically important compounds. Our current study identified a β-glucosidases from the *P. grandiflorus* plant that can convert PE into the more stable, smaller molecular weight, and more pharmacologically active PD, providing new insights for the analyzing the biosynthetic pathway of triterpenoid saponins in *P. grandiflorus*. Our findings will also lay a foundation to increase PD accumulation through molecular biological approaches, improve the quality of medicines, and expand the resources of PD.

Light is an important environmental factor for plant growth and development. It provides essential light energy for plant growth and acts as an environmental signal to regulate plant development. The downstream regulatory factors in the light signal transduction pathway mainly interact with specific cis-acting elements in the promoters of light-controlled genes, thereby up-regulating or down-regulating the expression of specific genes. The cis-acting elements present in the promoters of light-controlled genes are called light-responsive cis-elements (LREs; Hiratsuka and Chua, 1997). A previously study has shown that *SmCP*, the gene encoding *Solanum melongena* cysteine proteinase, showed maximum expression in the dark; the correlation between binding activity and expression suggests that the regulation of *SmCP* is accomplished by binding to the G-box in its promoter region (Xu et al., 2003). Different flanking sequences around the light-responsive G-box core can mediate induction or inhibition (Hudson and Quail, 2003). The study of a GT element showed that it can activate the expression of the rice *phyA* gene in the dark (Dehesh et al., 1990) and that it has two functions of positive and negative regulation (Mehrotra and Panwar, 2009). The AT-rich sequence of *oat* as a negative regulatory element can reduce the expression of light-regulated *PHYA* genes under light conditions (Nieto-Sotelo et al., 1994). Our study found that the Pgβ-glucosidase gene showed an upward trend after 6:00 pm and peaked at 12:00 pm Combined with promoter sequence analysis, the finding revealed certain light-responsive elements such as AE-box, Box 4, GT1-motif, MRE, TCCC-motif, and TCT-motif (Supplementary Figure 6). Our results suggest that the gene might be regulated by light and its expression enhanced in dark environments.

In conclusion, we have successfully cloned and characterized the β-glucosidase from *P. grandiflorus*. We verified its function and provided a new perspective for analyzing the biosynthetic pathway of oleanane-type triterpenoid saponins in *P. grandiflorus*. Moreover, we found that the expression of this gene might be regulated by light, which contributes to the further study of its molecular biology. Our findings provide direction for the molecular breeding of *P. grandiflorus* and the improving of the quality of medicinal materials. Future work should investigate the function of β-glucosidase in the plant body and attempt to regulate the conversion of PE to PD in *P. grandiflorus*.

## Data Availability Statement

The original contributions presented in the study are included in the article/Supplementary Material, further inquiries can be directed to the corresponding author.

## Author Contributions

SX, XS, ZW, LW, and WJ designed the project and/or conducted aspects of the experimental work. XS, ZW, FM, XY, YL, JW, and XG conducted the experiments and the collection of electronic resources. SX, SG, and DP supported this work financially and participated in its planning. XS, SX, and LW wrote the manuscript. All authors contributed to the article and approved the submitted version.

## Funding

This work was supported by the National Natural Science Foundation of China (Grant No. U21A20406), the NSF of Anhui Province (Grant No. 1908085MH268), the Anhui University Collaborative Innovation Project (Grant Nos. GXXT-2019-043 and GXXT-2019-049), the Foundation of Hunan Key Laboratory for Conservation and Utilization of Biological Resources in the Nanyue Mountainous Region (Grant No. NY20K04), and Key Natural Science Research Projects in Anhui Universities (Grant No. KJ2021A0676).

## Conflict of Interest

The authors declare that the research was conducted in the absence of any commercial or financial relationships that could be construed as a potential conflict of interest.

## Publisher’s Note

All claims expressed in this article are solely those of the authors and do not necessarily represent those of their affiliated organizations, or those of the publisher, the editors and the reviewers. Any product that may be evaluated in this article, or claim that may be made by its manufacturer, is not guaranteed or endorsed by the publisher.

## Acknowledgments

We would like to thank the group of Xiaoya Chen from the CAS Center for Excellence in Molecular Plant Sciences, Shanghai Institute of Plant Physiology and Ecology, for donating the pET-32ɑ(+) vector and *E. coli* BL21(DE3). We also thank Qian Shen from Shanghai Jiaotong University for donating seeds of *N. benthamiana* and the pBI121 vector, and Zhenbang Liu from the Division of Life Sciences and Medicine, University of Science and Technology of China for helping in confocal laser scanning.

## Supplementary Material

The Supplementary Material for this article can be found online at: https://www.frontiersin.org/articles/10.3389/fpls.2022.955628/full#supplementary-material

## Footnotes

^1^ http://www.expasy.ch/tools/

^2^ https://meme-suite.org/meme/tools/meme

^3^ http://www.ncbi.nlm.nih.gov/cdd/

^4^ http://www.cbs.dtu.dk/services/TMHMM-2.0

^5^ https://www.genscript.com/psort.html

^6^ https://npsa-prabi.ibcp.fr/

^7^ https://www.ebi.ac.uk/pdbsum/

^8^ https://swissmodel.expasy.org/

^9^ http://bioinformatics.psb.ugent.be/webtools/plantcare/html/
