# Supplementary material for: Molecular Cloning and Functional Characterization of a β-Glucosidase Gene to Produce Platycodin D in Platycodon grandiflorus
Source: Front Plant Sci. 2022 Jul 4;13:955628. doi: 10.3389/fpls.2022.955628 (PMC9289601; doi:10.3389/fpls.2022.955628)
Supplement: Supplementary file 1 [file Data_Sheet_1.docx]

Supplementary Material

# Supplementary Tables

**Supplementary Table 1.** β-Glucosidase gene sequence information.

| Gene | Sequence | |
| --- | --- | --- |
| β-glucosidase gene | | ATGAGTATAACGGAGAGGAAGCGAGAAATTGAGCAAGAATGGAGTAGTTGCATCTCCAACAACAACAACCCACTCTGTCGAGCCAACTTCCCCCCAAACTTCCTCTTTGGCGTCGCCACTTCTGCTTATCAGGTTGAAGGAGCTTGCAATCAGGGTGGTCGAGGTCCTAGCACTTGGGATGCTTTTTCACATACTGAAGGCAAGATATGTGATGGAAAAGATGCAGATATTGCAGTTGATCAATACCATCGCTACAAGGAGGACATTGACCTTATTGCCAAATTGGGATTTGGTGCTTATCGGTTTTCTATATCTTGGTCACGTATATATCCTGATGGCTTGGGAACCAAAGTCAATGATGAAGGGATAGCGCATTACAATAATTTGATTAATTCTCTTCTTGAGAAGGGAATCGAGCCTTATGTAACTTTATACCATTGGGATCTTCCATTGCATCTTCATGAGTCACTCGGAGGGTGGTTAAATGAGTCAATTGTAAAGTGTTTCGCCATCTATGCGGAAACTTGCTTCGCCAGTTTTGGTGACAGAGTGAAGAAGTGGATCACGATAAATGAGCCTCTTCAGACAGCAATCAATGGATATTGTACTGGTGTATATGCACCTGGAAAAAATGAAAATCCATCGACTGAACCTTATTTGGCTGCTCATTACCAGCTCTTGGCCCATGCTGAAGCTGTTTCGATATATAGAGAAAAATTTAAGGAGCAGCAAGGTGGAGAAATAGGCTTGGTGGTGGATTGTGAATGGGCTGAGCCTTTGTCAGATAATATAGAAGATAAAAATGCTGCAGCAAGGCGTCTCGATTTTCAGCTTGGATGGTATTTGGATCCAATATTTTATGGAGACTACCCTGAATCTATGCAAGAGAGACTTGGAGATTCGCTTCCAATATTCTCCCAGAAAAATAAAGATTTACTCAGGAATTCAGTGGACTTTGTTGGCCTGAATCACTATACCTCAAGGTTTGTGAGACAAGGAACTGATAAACAAAATGAATACTATAGAGTACAAGAGGTGGAAAGAATTGCGGAATGCGAAGGAGAAGCAATTGGTCCGAAGGCAGCTTCACCTTGGCTTTACATTGTTCCTTGGGGCATTCAAAAAGTTCTCAATTACATTGCTCAGAGATACAATAATCCACCAATATATGTTACTGAGAATGGGATGGACGATGAAGACAATGATGTTTCTCCCCTCCATGAGATGCTGGATGACAAATTAAGAGTTTCTTACTATAAGGGCTACCTAACTGCTGTTGCTCAGGCTGTCAAGGATGGAGCAGATGTGAGGGGTTACTTTGCATGGTCATTGTTGGACAACTTTGAGTGGCAGTATGGTTATACAAAGCGTTTTGGTTTGATATACGTGGACTTCAAAAATGGGCTCACTCGGCATCTAAAATCGTCTGCTTATTGGTTCGTGCGATTCTTGAAAGGTGGAGAAGGGAAAAGTGGCAAAGAAGAGTAG |

**Supplementary Table 2.** β-Glucosidase gene promoter sequence information.

| Gene | Sequence |
| --- | --- |
| Promoter of β-glucosidase gene | AGTTTAGGGACCAAATTGTATATTAAACCAAAATAAAATTGAATGATCAATAATATTATATTTGGAATATTTTAGATTATTATAAATCTAGCAAATCTTGAATGTATAATAAATTTAGATCTGAAATAAATAGGTAAAAGAAATACCTCATGGGCTGAAATTAGTTATCATGTAGTGAATTCTGACTTTTAATAATCGTTTTCTTTTCCTAAGACGAACATCATTTTAAAATTAAAAAAATTTCAAAGAACAATTGAAGTATTGTTTCAATGGAAAAGTCAAACATAATTTCTTACCGAATCCTTTCATTTTACAATGAGATCTGAGAAGGCGTAGAGGAATTTGGGGATACAGGGTGGAGGCTATTTTTGGGTATTTAACTATTTATATTTGGTTAGTGTAGGTCGGAATGTTGGGCCCTATTTATTAGGGTAATTCAATTTGGGTCGGACTGATTTTTATTAATTTGAATTAGGGAGAATTTTAGGCGGTCACCTATGGATAATTTTCAACCGCTTTATACCTATATTCTTTCGTAATTAAAAAAAATGCACATTGTGTAAATATATGCACAAAATCATGCATCGCTATGCACAAATTGTGCATCACGATGCACAAAATTTTATAAAAAACACTATATGTGTGTTAGAGTAAAACTTTAGATTAATGTTTCGGTTGGTAGAAATAAAGTAAATGAGAGTTATAGAGGTAACTGACTTTATATATGGAAAGAGTGGGTATTAGGTTTTCAATTTGGCCCCAAATTAATTGGATGTATTTAATTTGAGTCATATTTGTTACTAGCCCGTTAATAATTTGATTCGTTTTTCAATTTTCAATGTATTTAATTCTTTTGTCCATCATCCATGTTCTCTTGTTTCTTTTTGATTTTCTTTTTTTTTGTTGTTGAACTTGTGATTACGACTACTTAAAAAATAAAAAATACATTCATGTTGTTTGATTTCCATAAATACAGTGCCTTCAATCCTTTGGGGGTGAAAATTGAAAAACTCCGTTTACTATTACAACCTCTCTCTGGGTTGAAAATTTCCTTTAATCTTTACAAAAAAAATGAATTAAAAAATTTAACTTTACTGAATTAAAGAAGAAGGCAAGGAGTAGTTTGGGTATTTTTTTTAAAAAATAGAAGGAAAATTTAACGTTAACGACTTGAAGGGAAAATCTCACAAAAAAATGTAAATTAAGTGACCGAAATAAATTATTTTGATAAGAGAGACTAAATACATAGATTTGTTAAAGTGGAGAGACTAAAAAATTATTTAATTTTTTTTTCCATTGAGAAGCGTGATGCATATGATCAGAGAGACAATTGCCATGTAATGAAGGTCTCTGTCATGTCACGGTTCAATGCTTCACTTTTTCTTTGCGGATTATTATTTACACCCTCTTAATCTTTAAGAACACCATTTCTCTAAATTCCCCTCAATTTTTCATTTTATTCCACCAAATTTATTTTGAAGACACACACACAAGAGTGAAGAAACCTTAGCATCATCTCAAAACTCTACTCCCAAAGCAAGCTATCTGTAAAAATTGGTAGGGTTAAGTTAGATACGATTTTCTTTACAATTTAAGTTTAAAATTGGTGATTTATGTAAAAAAATTACATTTTAAGTTAAATTTCGAAGTGAACTAGTCTATATTGTTCGAACATAGTTGATTAAACACTTTTATTGTTGTTTGATCATTACCAACTAAACTTGTTCGATCGATACTCGACCAAACATCACTAAAAAGAATAAAGCTAACAATGTTCGGCCATCAATAATAAAACACTGGTAAAGAGTGTTCGATCACCACTGATAAACACCAATAAAAATAAATATTTTAATTTTTGTAATGATATTTTTGTAATAATGAAAGTGGCCTACTTGTAGAGAAGCGGGGCGAATAGCAATTCTCTTCTTCTTTGGGACGTACGGACACTTCTTTCCCCTCCATCCGTTTTCCCCCATTTTCGTAACAGTTAATTGCCGATT |

**Supplementary Table 3.** Primers sequences information.

| Name | Sequence (5'-3') | Function |
| --- | --- | --- |
| β-glucosidase-F | ATGAGTATAACGGAGAGGAAGC | For PCR |
| β-glucosidase-R | CATCTCTTCTTTGCCACTTTTCCC |  |
| 18s rRNA-F | TGACGTTGAAGCTCGCAAGTACAC | For qPCR |
| 18s rRNA-R | TCATCAGGCAAGGCAAGAAGTTCC |  |
| β-glucosidase-1-F | GGAAAGAATTGCGGAATGCGAAGG |  |
| β-glucosidase-1-R | TGCCCCAAGGAACAATGTAAAGCC |  |
| β-amyrin-F | TTAAGGTGGCTGATGGTGGTAATGATC |  |
| β-amyrin-R | AGCGGTTGTTCCAGAAGTCTTGAC |  |
| β-glucosidase-2-F | GTCGACATGAGTATAACGGAGAGGAAGC | For subcellular localization |
| β-glucosidase-2-R | ACTAGTACCTCTTCTTTGCCACTTTTCCCT |  |
| β-glucosidase-3-F | ATAGCTGATGAGTATAACGGAGAGGAAGC | For preparation of recombinant β-glucosidase |
| β-glucosidase-3-R | CTCGAGCATCTCTTCTTTGCCACTTTTCCC |  |

**Supplementary Figures**


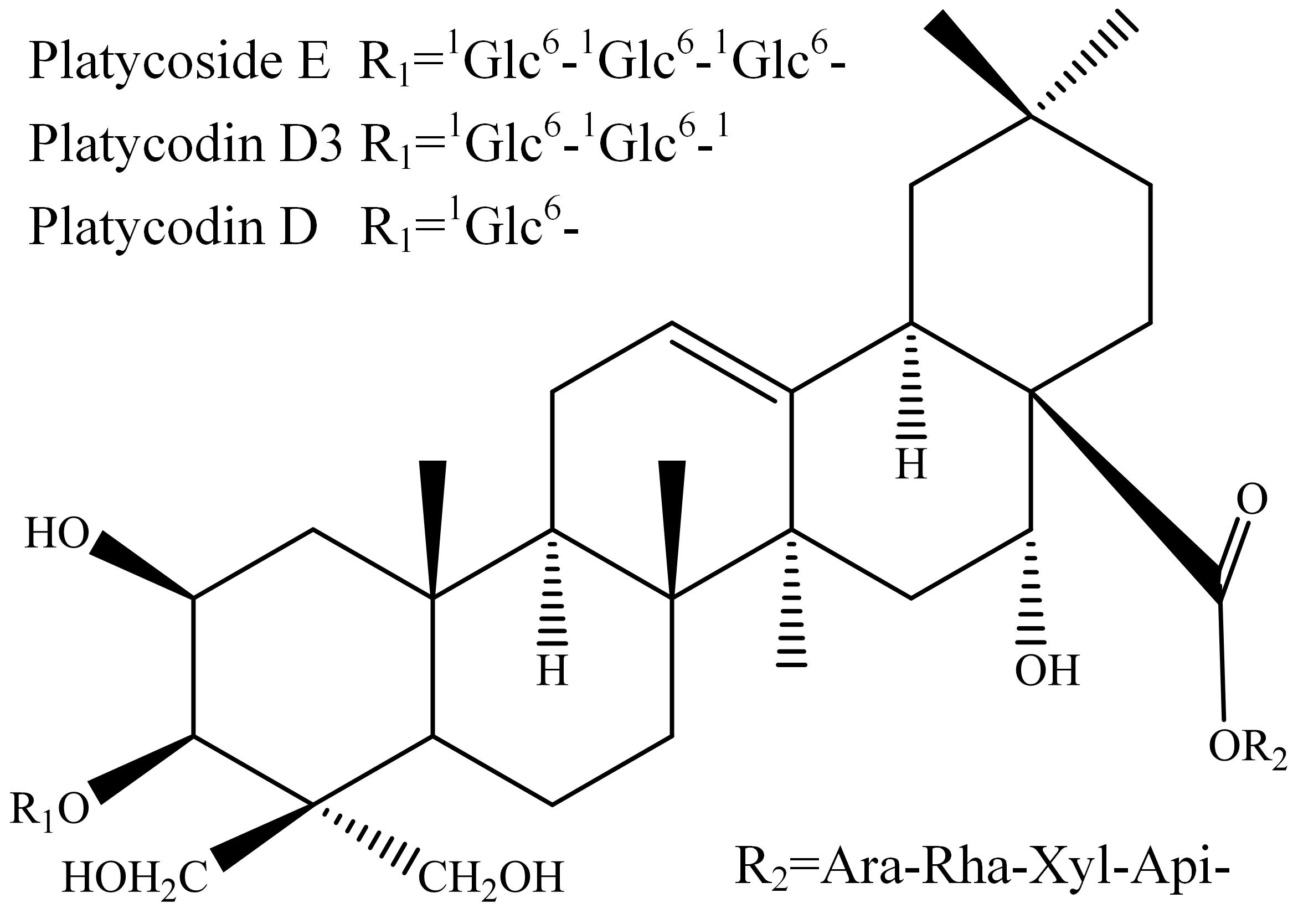


**Supplementary Figure 1.** Chemical structural formulas of platycodin D and platycoside E.


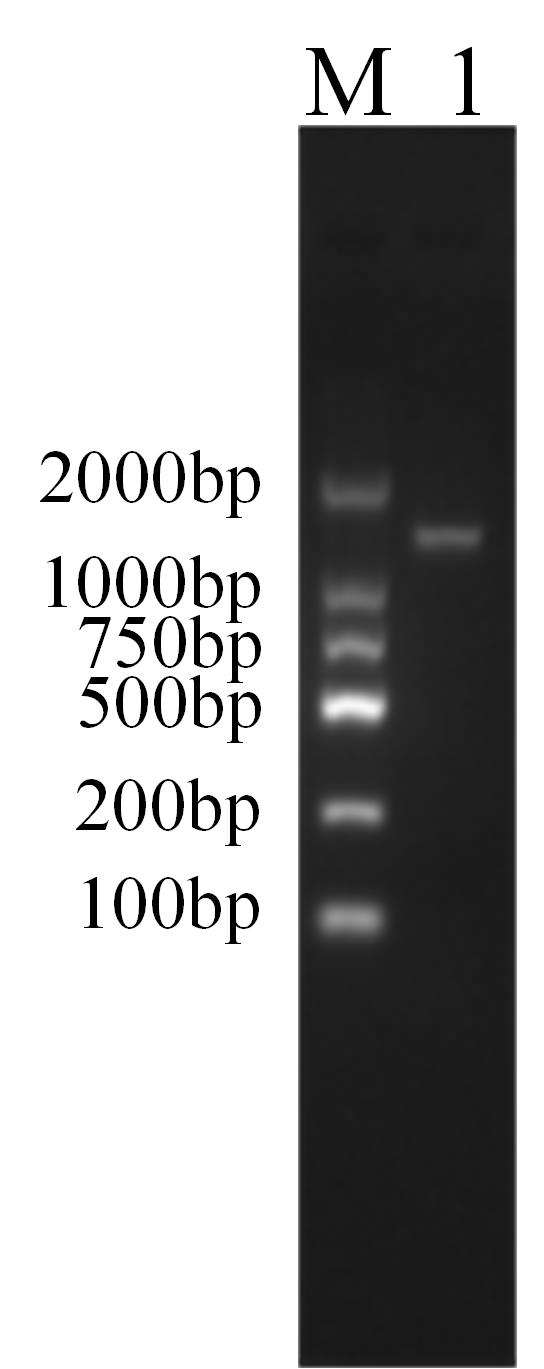


**Supplementary Figure 2.** PCR amplification product of gene fragment encoded Pgβ-glucosidase.


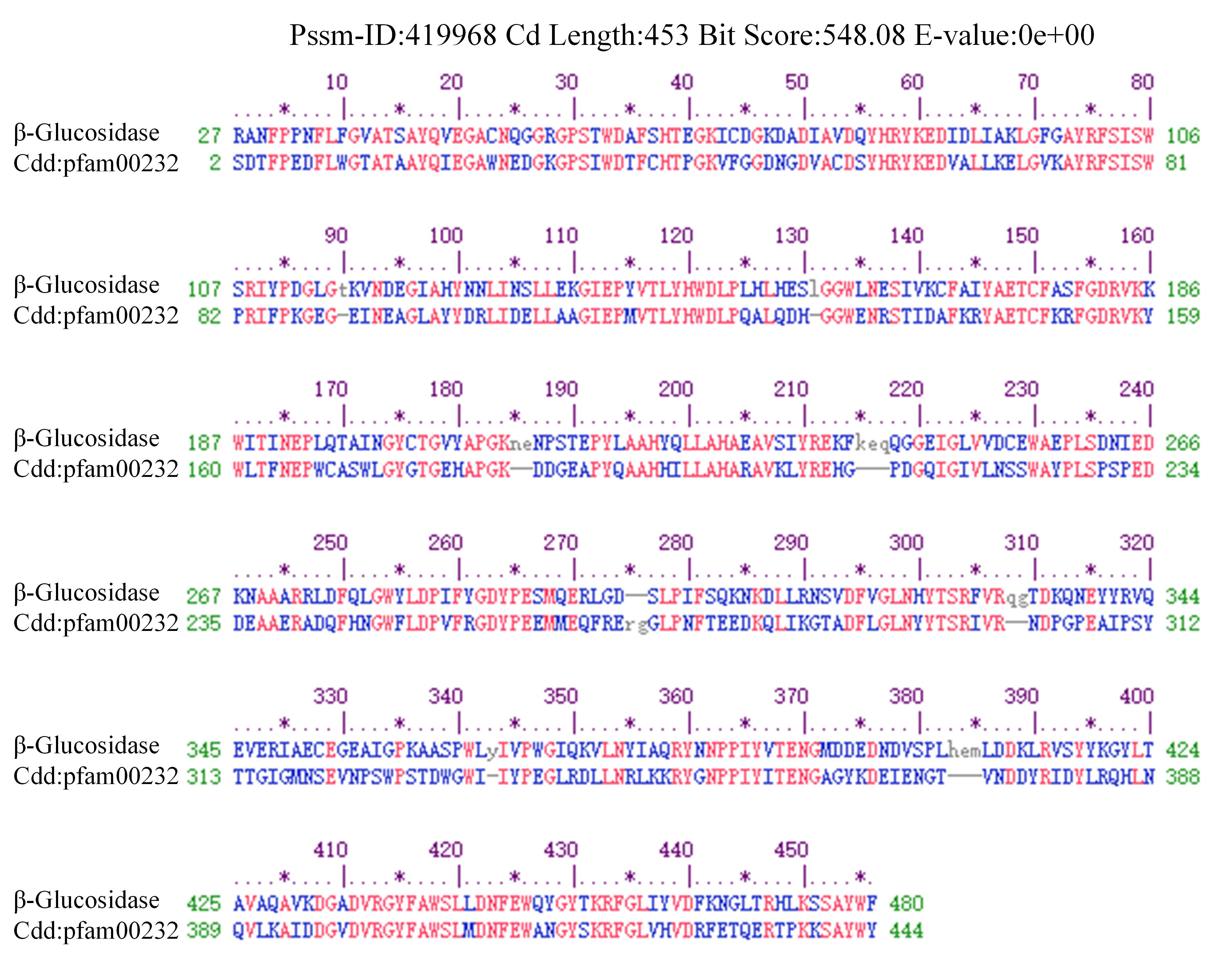


**Supplementary Figure 3.** Comparison results of Pgβ-glucosidase protein in NCBI conserved domain database.


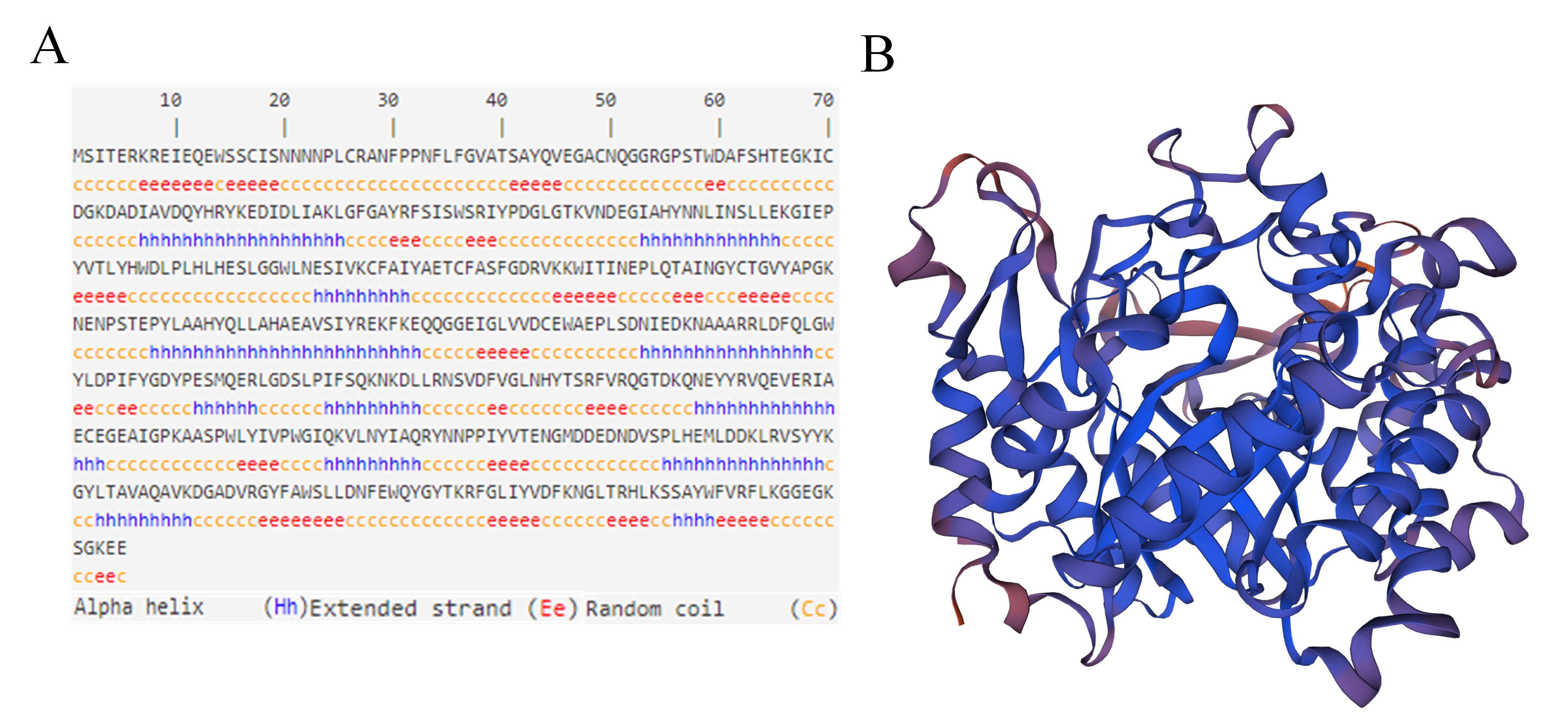


**Supplementary Figure 4.** Secondary structure and tertiary structure model of Pgβ-glucosidase. **(A)** The predicted secondary structure of Pgβ-glucosidase. **(B)** The predicted tertiary structure of Pgβ-glucosidase.


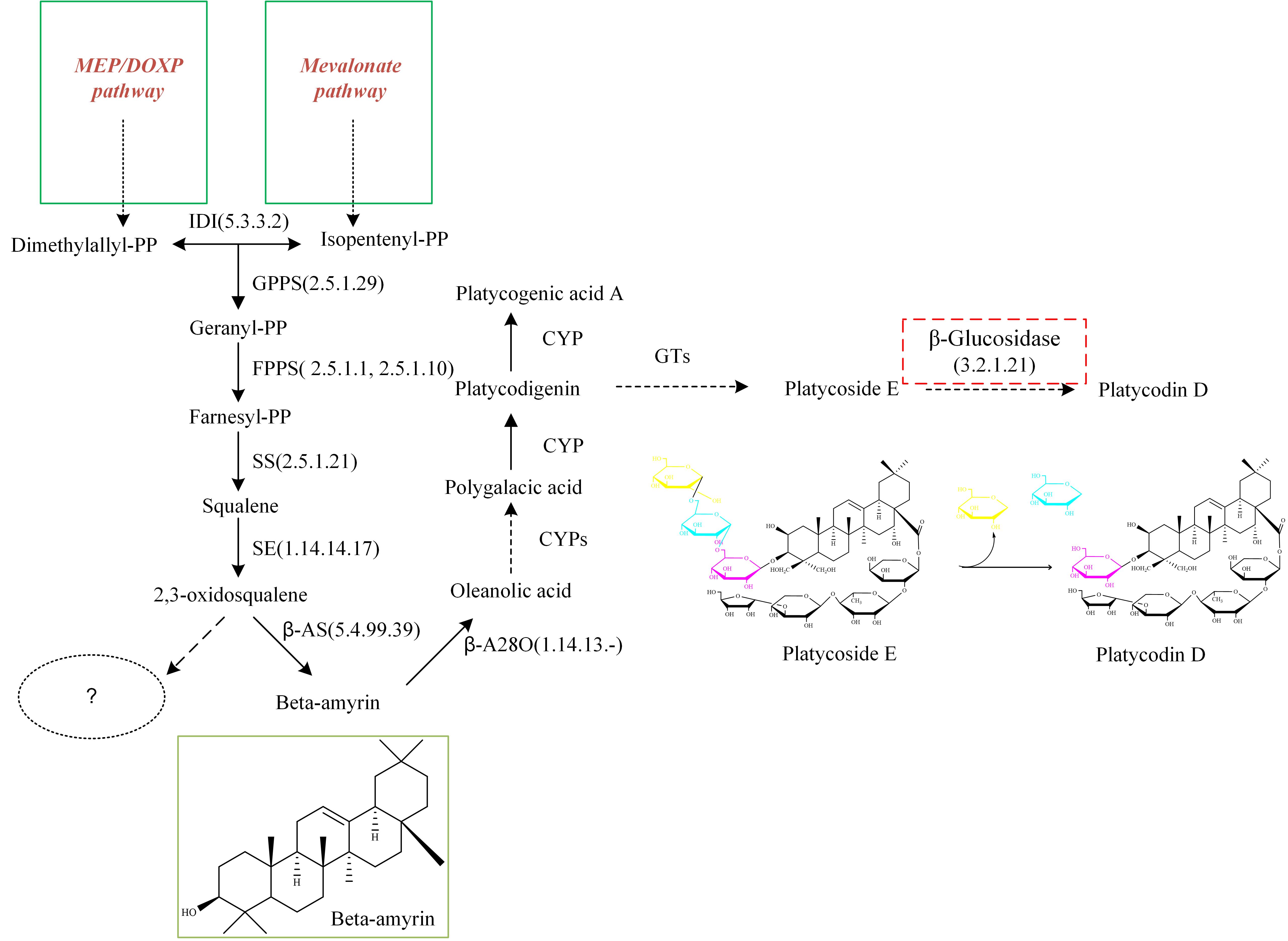


**Supplementary Figure 5.** Biosynthetic pathway of oleanane-type triterpenoid saponins from *Platycodon grandiflorus*. The dotted line represents the multi-step enzymatic reaction, and the red dotted box is the predicted functional gene in this paper. IDI (isopentenyl-diphosphate Delta-isomerase), GPPS (geranylgeranyl pyrophosphate synthase), FPPS (Farnesyl-diphosphate synthase), SS (squalene synthase), SE (squalene epoxidase), β-AS (beta-amyrin synthase), β-A28O (isolate CYP716A140 beta-amyrin 28-oxidase), GTs (Glycosyltransferase).


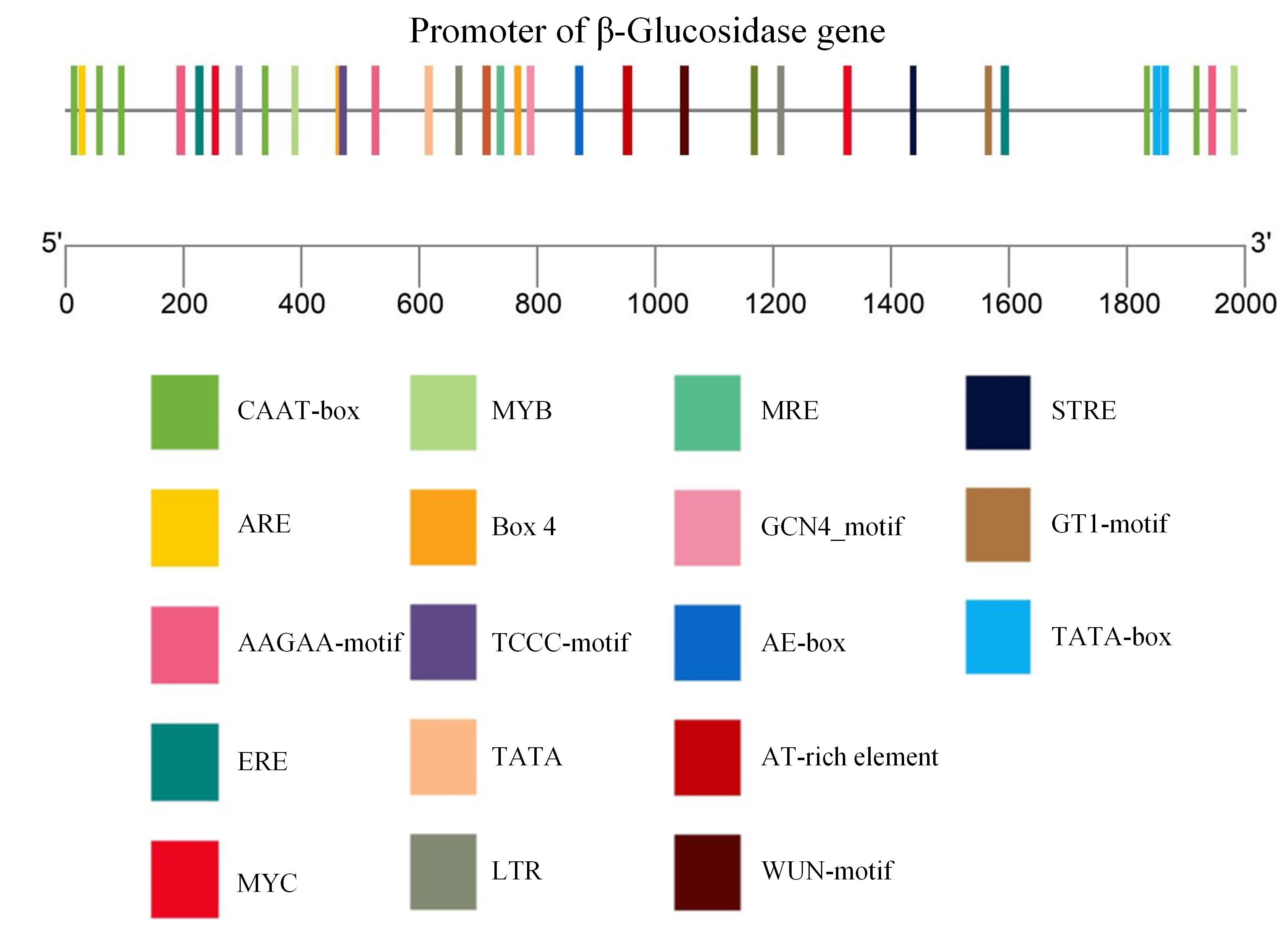


**Supplementary Figure 6.** Promoter sequences analysis of Pgβ-glucosidase encoding gene to predict cis acting elements.
